# The Effects of Hand Preference on Measures of Psychological Well-Being in a Sample of Older Adults in the Kingdom of Saudi Arabia

**DOI:** 10.7759/cureus.39058

**Published:** 2023-05-15

**Authors:** Khulud K. Alharbi, Tamara A. Hafiz

**Affiliations:** 1 Department of Health Services Management, Faculty of Public Health & Health Informatics, Umm Al-Qura University, Makkah, SAU; 2 Department of Health Promotion and Education, Faculty of Public Health & Health Informatics, Umm Al-Qura University, Makkah, SAU

**Keywords:** saudi arabia, wellness, quality of life, risk factors, mhqol, prevalence, left-handedness

## Abstract

Background: Generally, being left-handed has been linked to poorer mental health and quality of life. However, given that few studies have investigated these links in Saudi Arabia and that the prevalence of mental illness in the general population is rising, it is important to explore whether left-handedness can be considered a risk factor in a sizable, general population.

Aim: To investigate whether left-handers experience psychological well-being or good quality of life.

Methods: A cross-sectional study among adults in Saudi Arabia was conducted from March 6, 2022 to February 27, 2023.

Results: The study included (N = 2862) respondents who met the inclusion criteria, with an average age of 28.95 years. Left-handed individuals made up 31.7%, whereas right-handed individuals made up 60.3%, and ambidextrous individuals made up 7.9% of the population. Using the Mental Health Quality of Life questionnaire (MHQoL-7D) scoring manual, the quality of life for both left-and right-handers was evaluated. Those who were right-handed had a higher quality of life than those who were left-handed. Multivariate Analysis of Variance (MANOVA) was conducted, and the findings showed that neither the left-handed nor the right-handed group significantly differed in their levels of poor quality of life or psychological well-being.

Conclusion: Using the left or the right hand had no effect on one’s quality of life or degree of well-being. Further studies with a larger sample size are needed to examine this result in more detail.

## Introduction

People who are distinctively different have always aroused astonishment and attention [1]. The profile of the lateral organization, which is a particular mix of functional asymmetries of the brain hemispheres (mental, motor, and sensory), is one of these unique characteristics that make a person stand out from others [1]. The natural or biological propensity to use one hand more often than the other when executing tasks is known as handedness [2]. If a person uses their right hand more frequently than their left, they are said to be right-handed, and vice versa for left-handed people. Thus, left-handedness is described as the preference for the left hand over the right hand when carrying out daily activities like writing, throwing, and brushing teeth [2]. There are numerous definitions and classifications of handedness. Most people define handedness as the hand that one uses for writing. Also, some researchers claim that the right hand is the fastest and most accurate hand. Others consider it to be the preferred hand, despite its limitations. While some people almost exclusively use their right or left hands, others alternate between the two depending on the activity, and some typically complete chores with either hand (meaning that they are good at using both hands) [3]. However, according to earlier data, being left-handed is not prevalent; an outdated statistic estimates that 8-15% of persons globally are left-handed [2]. According to the findings of one study, 90% of people use their right hand for most everyday tasks, whereas 10% use their left hand [3].

Right-handedness was the norm for millennia, whereas left-handers were the world’s largest minority and were shunned culturally, socially, and even linguistically for millennia [4]. Various studies on the biological effects of departures from the “normal is right-handed” dominance pattern have emphasized that these abnormalities may explain the greater occurrence of developmental, cognitive, and mental illnesses among left-handed people [5,6].

Because left-handed people are a minority in society, they do not receive a lot of attention [3]. For instance, manufacturers always produce goods in accordance with the population’s preferences, and most items are only designed for the right-handed, forcing the left-handed to use them. Examples include door handles, computer keyboards, cufflinks, two-wheeled garage door opener accelerators, and pencil sharpeners [3]. These affordances may significantly affect people’s levels of wellness and mental health. Moreover, some people still view left-handedness as deviant, while others see it as a sign of genius [1]. This attitude, as well as others like it, may also have an impact on one’s level of mental wellness. Yale researcher Jadon Webb and his colleagues discovered that among those with mental illnesses, those with psychotic disorders like schizophrenia are much more likely to be left-handed than those with mood disorders like depression or bipolar syndrome [7]. As Webb et al. (2013) state “Finding biomarkers such as this can hopefully enable us to identify and differentiate mental disorders earlier, and perhaps one-day tailor treatment in more effective ways” [7].

The ability of each of us as individuals and as a species to think, express ourselves, engage in social interaction, make a living, and enjoy life depends on our mental health. The World Health Organization defines mental health as a condition of well-being characterized by self-awareness, the ability to manage the pressures of daily life, the capacity for productive work, and the capacity to contribute to one’s community [8]. As a result, encouraging, safeguarding, and regaining mental health can be seen as crucial concerns of people and societies everywhere in the world. Therefore, the crucial query regarding the history of hand use is, “Are there actually differences between the hands?” Are there significant distinctions between the hands that could have an adverse psychological effect and prevent wellness and mental health, or disrupt daily activities, or affect productivity at the individual or societal level? This lack of resolution means that the phenomenon being studied is still not fully understood. Many concerns surrounding left-handedness, including the causes and the specific psychological and physiological factors, remain unaddressed. While there is limited research on interventions specifically targeted at left-handed individuals, there are several strategies that could help improve the psychological well-being of these individuals. One approach could be to address the social stigma surrounding left-handedness through educational programs and public awareness campaigns. Another strategy could be to offer counseling services that are tailored to the unique experiences of left-handed individuals. Additionally, cognitive training programs that focus on improving spatial reasoning skills in left-handed individuals could be developed to address cognitive differences. Therefore, the current study was conducted to investigate whether left-handers experience psychological well-being. Understanding the relationship between left-handedness and psychological well-being can have important implications for parents, educators, and mental health professionals.

 Aim of the study

The study aims to measure the prevalence of left-handed preference and determine whether left-handedness can affect the quality of life and wellness. Additionally, the roles of demographic factors and health status as determinants that may affect the mental quality of life and wellness across adults in Saudi Arabia were investigated.

## Materials and methods

To assess the impact of left-handedness on psychological well-being among the adult population across all the regions in Saudi Arabia, a cross-sectional web-based survey was done from March 6, 2022 to February 27, 2023.

The entire adult population of Saudi Arabia throughout all Kingdom regions (western, northern, southern, central, and eastern), comprising 13 provinces and regions, was included when calculating the sample size using the Raosoft Sample Size Calculator [9]. According to the General Authority for Statistics’ 55th statistical yearbook and the updated mid-2020 report, this population has 23,735,248 people between the ages of 18 and 60. The standard deviation was set at 1.96 for the 95% confidence interval, the margin of error was 5%, and the expected response was 50%; thus, 385 samples were needed as a minimum.

Individuals between the ages of 18 and 60 years are considered to be within the inclusion age range, while those under 18 and those over 60 years are considered to be outside the eligibility range (Table 1).

**Table 1:** Inclusion and Exclusion Criteria.

Inclusion criteria	Exclusion criteria
Saudi Adults	Non-Saudi adults
Being over 18 years old	Under 18 years, or greater than 60 years
No suffering from any mental or psychological condition	Suffering from any mental or psychological conditions

Data collection tools

Data were collected through structured self-questionnaires prepared in Arabic. The responders took around 3-6 minutes to complete the questionnaire. A purposive convenience sample was recruited by SMS/WhatsApp distribution. A non-probability sampling design was used in order to access the largest number of the target population. Written consent was given in the first section of the online questionnaire to all participants prior to filling out the questionnaire. The questionnaire included: 1. Socio-demographic characteristics, including age, gender, region of residence, marital status, occupation, educational level, and one more question about the presence of chronic diseases. 2. Hand distribution to the participants-Handedness preference was assessed using a modified questionnaire based on the Edinburgh Handedness Inventory (Oldfield, 1971). The questionnaire was translated into Arabic and included 8 items: (1) writing, (2) drawing, (3) throwing, (4) holding a glass/cup, (5) brushing teeth OR (6) spoon, (7) using an eraser on a paper (8) removing a paper/card from the table or desk. 3. Inheritance pattern of handedness among the study participants. 4. The questionnaire on Mental Health Quality of Life (MHQoL) [10].

The mental health quality of life questionnaire (MHQoL)

The Mental Health Quality of Life questionnaire (MHQoL) is a self-administered, standardized measure of the quality of life that has been created for use in individuals with subclinical and clinical mental health issues as well as across all mental health service types [10]. The questionnaire was also used with the general population e.g. in a cross-sectional study of 192 Korean pay workers [11]. The MHQoL is divided into two components: the MHQoL-7D descriptive system and the MHQoL-VAS visual analogue scale.

The MHQoL-7D consists of seven questions, each with four response options ranging from very satisfied (score = 3) to very dissatisfied (score = 0), covering seven aspects (self-image, independence, mood, relationships, daily activities, physical health, and future). An overall index score can be calculated by summing the scores of the seven questions, which ranges from 0 to 21, with higher scores indicating better quality of life. 

The MHQoL-VAS records the self-esteemed general psychological well-being of the respondent on a horizontal scale ranging from zero ("worst imaginable psychological well-being") to ten ("best imaginable psychological well-being") [10].

Reliability of (MHQoL)

The overall MHQoL-7D's Cronbach's alpha coefficient was 0.85. The item-total correlations varied from (0.48 to 0.71), and without lowering Cronbach's alpha, none of the items could be removed. The entire MHQoL-7D had a test-retest reliability of 0.85 as determined by ICC. For each item, the ICCs ranged from (0.51 to 0.77) [12].

Data management

All tests were executed using SPSS software, version 25.0 (IBM Corp., Armonk, NY). Where all demographic information, and items, underwent a descriptive analysis in the form of frequency and percentage. A series of Pearson correlations were performed between all of the dependent variables. The Multivariate Analysis of Variance (MANOVA) test was conducted to test the hypotheses for left-handed people and quality-of-life scores. The hypothesis was that there would be mean differences in the dependent variables (quality-of-life score and psychological well-being) for the three levels of independent variables (left, right, and ambidextrous) [13-14]. The P-value was calculated using the MANOVA test, and a P-value of 0.05 or less was regarded as statistically significant. Finally, tables and graphs were used to present the study's findings.

Ethical consideration

Ethics were approved by the Umm Al-Qura University Bioethics Committee; approval no. (HAPO-02-K-012-2022-11-1230). Participants also received an online information letter, which served as the first cover page before starting the online survey, emphasizing that participation was entirely optional and that participants might withdraw at any time, for any reason.

## Results

With an average age of 28.95 years, a standard deviation of 8.85, and a range of ages from 18 to 60, the study comprised 2862 respondents (N = 2862) who matched the inclusion criteria. The majority (72.2%) of these respondents were in the 18-30 age range. Most participants (78.9%) were female, married (33.4%), and lived in the west, central, south, or north regions: 44.5%, 22.4%, 17.8%, 9.5%, and 5.7%, respectively. Almost (34.6%) were registered in a primary, middle, or high school, while almost more than half (60 %) were still in a Bachelor's degree, finally 428 (15%) had a chronic disease (Table 2).

**Table 2:** General characteristics of the participants. ^1^Some participants had more than one chronic condition, this includes 155 (5.6%) who had asthma, 120 (4.3%) hypertension, 113 (4.1%) diabetes, 89 (3.2%) mental or psychological illnesses, 70 (2.5%) high cholesterol, 32 (1.2%) cardiovascular disease, 19 (0.7%) kidney disease, and 8 (0.3%) cancer.

Characteristics		Frequency (N)=2862	Percentage (%)
Age (years)	18–30 years	2065	72.2
	31–40 years	419	14.6
	41–50 years	257	9.0
	51–60 years	121	4.2
Gender	Male	605	21.1
	Female	2257	78.9
Marital status	Married	955	33.4
	Unmarried	1907	66.6
Region	Western	1274	44.5
	Central	642	22.4
	Eastern	272	9.5
	Southern	510	17.8
	Northern	164	5.7
Educational level	Primary school	25	.9
	Middle school	91	3.2
	High school or diploma	874	30.5
	Bachelor’s degree	1716	60.0
	Postgraduate	156	5.5
Occupation	Governmental sector	492	17.2
	Private sector	323	11.3
	Retired	69	2.4
	Student	1328	46.4
	Do not work	650	22.7
Medical history of chronic diseases	None	2434	85.0
	Yes^1^	428	15.0

**Figure 1:**
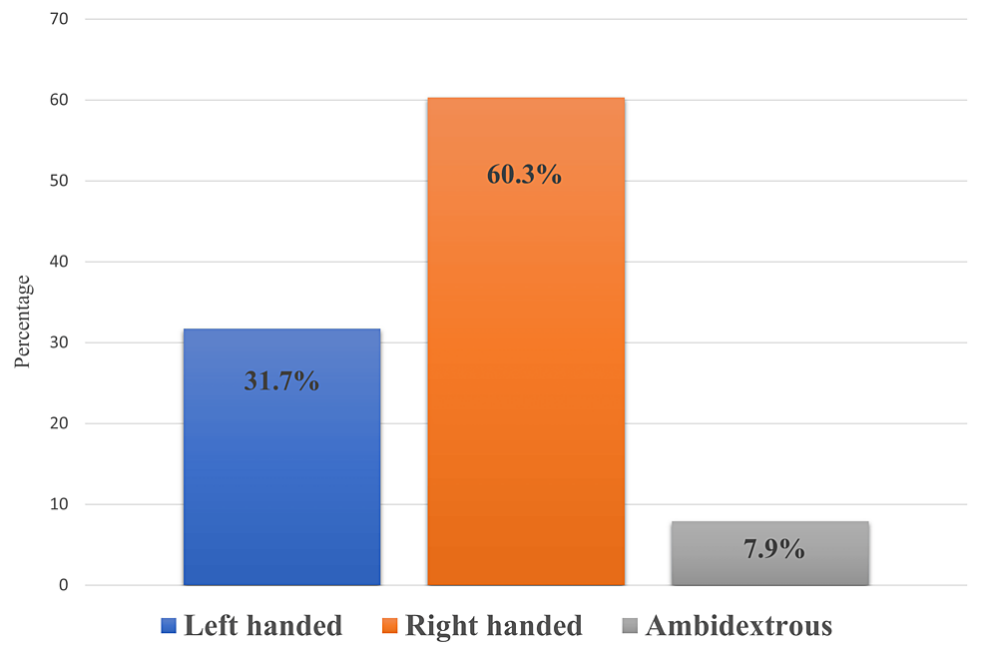
Classification of participants according to handedness distribution.

The distribution of handedness among the study population is depicted in Figure 1 as a percentage. Left-handed persons made up 31.7% of the population, or 908, while right-handed people made up the largest percentage with 60.3%, or 1727 people, and ambidextrous people, who were able to use the right and left hands equally well, had the lowest percentage, 7.9, with just 227 people participating (Figure 1).

According to the sociodemographic factors, there is a big variation in the distribution of individuals based on their hand preference. From Table 3, it is clear that most of the respondents were 18-30 years old, and they were divided into left-handed, right-handed, and ambidextrous groups of 26%, 40.15%, and 6%, respectively. Also, females took up a big part of the left-handed sample at 729 (25.47%) and the right-handed sample at 1346 (47.03%), with only 182 (6.36%) being ambidextrous participants. Finally, in the unmarried group, the big sample belonged to right-handed people with 1060 (37.04%), followed by left-handed people with 687 (24%), and finally, the ambidextrous sample represented 160 (5.59%). There is a variation in distributing the sample across regions; left-handed people were represented the most in the western, central, and southern regions (12.58%, 9.47%, and 4.09%, respectively). Right-handed people were mainly in the western, southern, and central regions (28.97%, 12.09%, and 11.01%, respectively). Finally, the ambidextrous group was mainly in the western with 2.97% and the central with 1.96%.

**Table 3:** Handedness distribution across sociodemographic factors. N= number

Characteristics N = 2862		Left-handed people N=908 (%)	Right-handed people N=1727 (%)	Ambidextrous N=227 (%)
Age (years)	18–30 years	744 (26)	1149 (40.15)	172 (6.01)
	31–40 years	100 (3.49)	291 (10.17)	28 (0.98)
	41–50 years	45 (1.57)	189 (6.60)	23 (0.80)
	51–60 years	19 (0.66)	98 (3.42)	4 (0.14)
Gender	Male	179 (6.25)	381 (13.31)	45 (1.57)
	Female	729 (25.47)	1346 (47.03)	182 (6.36)
Marital status	Married	221 (7.72)	667 (23.31)	67 (2.34)
	Unmarried	687 (24)	1060 (37.04)	160 (5.59)
Region	Western	360 (12.58)	829 (28.97)	85 (2.97)
	Central	271 (9.47)	315 (11.01)	56 (1.96)
	Eastern	98 (3.42)	152 (5.31)	22 (0.77)
	Southern	117 (4.09)	346 (12.09)	47 (1.64)
	Northern	62 (2.17)	85 (2.97)	17 (0.59)
Educational level	Primary school	14 (0.5)	9 (0.3)	2 (0.1)
	Middle school	32 (1.1)	50 (1.7)	9 (0.3)
	High school or diploma	274 (9.6)	518 (18.1)	82 (2.9)
	Bachelor’s degree	531 (18.6)	1064 (37.2)	121 (4.2)
	Postgraduate	57 (2.0)	86 (3.0)	13 (0.5)
Occupation	Governmental sector	118 (4.1)	335 (11.7)	39 (1.4)
	Private sector	122 (4.3)	175 (6.1)	26 (0.9)
	Retired	10 (0.3)	57 (2.0)	2 (0.1)
	Student	478 (16.7)	740 (25.9)	110 (3.8)
	Do not work	180 (6.3)	420 (14.7)	50 (1.7)
Medical history of chronic diseases	None	771 (26.9)	1474 (51.5)	189 (6.6)
	Yes	137 (4.8)	253 (8.8)	38 (1.3)

Table 4 displays the frequency of preference for using the hand by left-handed participants in various activities. The survey found that 885 out of 908 left-handed participants-or 97.5%- preferred to use their left hand for three tasks, including writing, drawing, and erasing paper. Cleaning teeth, combing hair, catching a ball, and taking cards off the table were performed by left-handers using their left hands 82.5 %, 87.4%, 82.6%, and 87.6% more frequently than their right hands, respectively. However, just 42.5% of left-handed people favored left-handedness when holding a spoon, whereas 57.5% chose right-handedness.

**Table 4:** Frequency of left-handed preference of some specific activities among left-handed participants.

Activities	Right hand	Left hand
Writing	23 (2.5)	885 (97.5)
Brushing teeth	159 (17.5)	749 (82.5)
Holding a cup	407 (44.8)	501 (55.2)
Holding a spoon	522 (57.5)	386 (42.5)
Brushing or combing hair	114 (12.6)	794 (87.4)
Drawing	23 (2.51)	885 (97.5)
Throwing the ball	158 (17.4)	750 (82.6)
Using an eraser on paper	23 (2.5)	885 (97.5)
Removing a sheet or card from a table or desk	113 (12.4)	795 (87.6)

**Figure 2:**
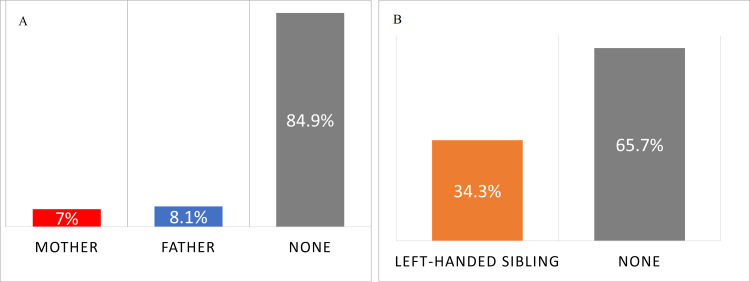
Percentage frequency of the inheritance pattern of handedness among the study participants. (A) Handedness of parents (B) Handedness of siblings

Figure 2A shows the distribution of left-handed parents as an inheritance pattern of handedness among the general participants: 200 (7%) mothers and 231 (8.1%) fathers. Figure 2B shows the distribution of left-handed siblings as an inheritance pattern of handedness among the general participants: 34.39% or 981 participants had left-handed siblings.

**Figure 3:**
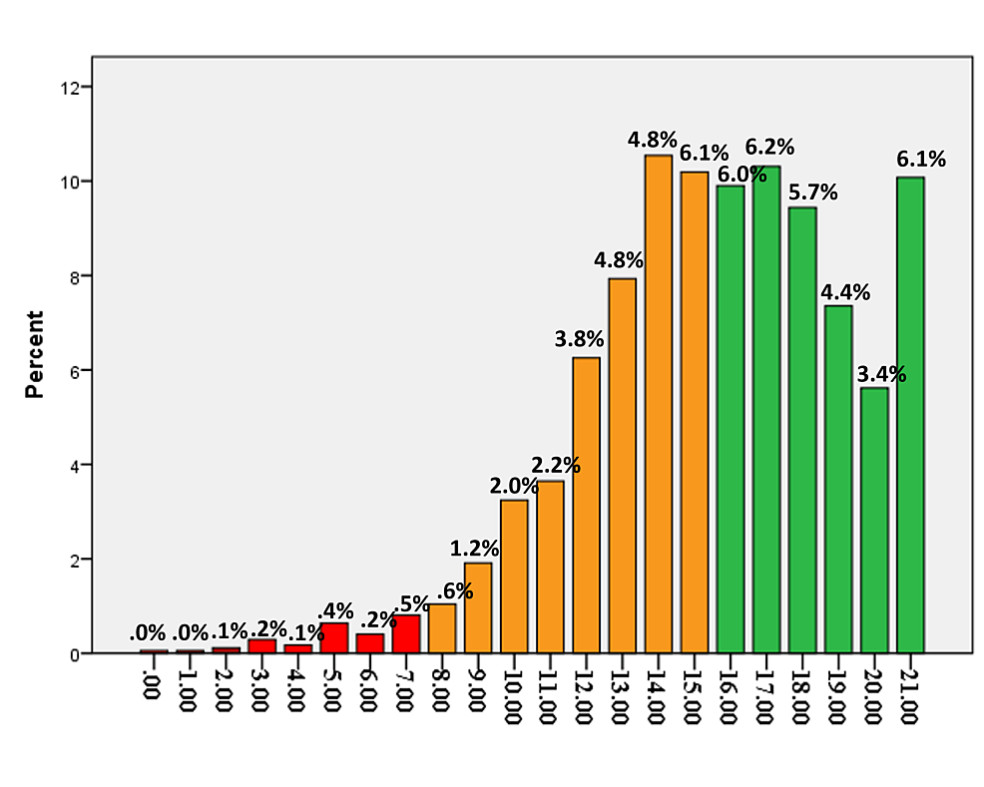
Overall MHQoL-7D score for right-handed participants.

**Figure 4:**
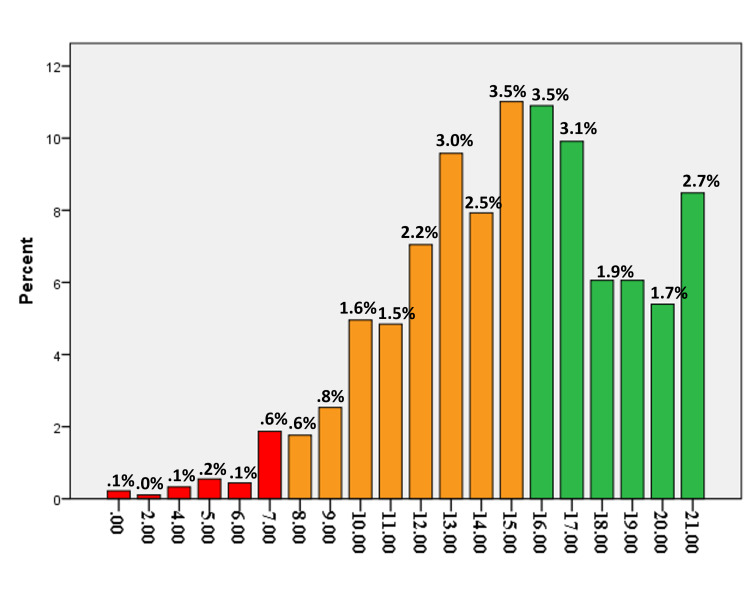
Overall MHQoL-7D score for left-handed participants.

The quality of life of both left-handers and right-handers, via the MHQoL-7D scoring manual, was examined in Figures 3, 4. They all enjoyed high levels of quality of life, though. Participants who were left-handed reported having a poor quality of life compared to right-handed people.

**Figure 5:**
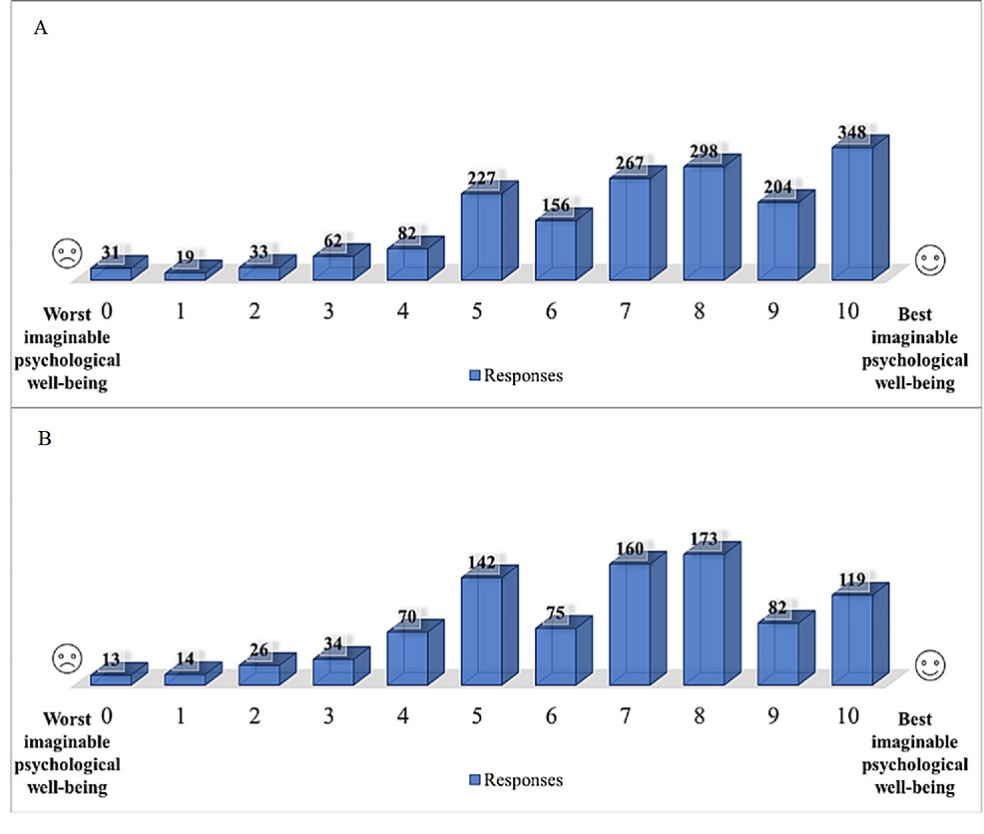
Psychological well-being for right- and left-handers. (A) Psychological well-being for right-handed participants, (B) Psychological well-being for left-handed participants.

The degree of psychological well-being for right-and left-handers is compared in Figure 5, where (10) depicts the best possible psychological state and (0) the worst possible psychological state. Each participant selected the number that best reflected their sense of psychological well-being. Figure 5 illustrates that despite the fact that the majority of participants belong to the right-handed category, the findings indicate that psychological well-being is prevalent in both categories.

Prior to conducting MANOVA for the quality-of-life scores and psychological well-being, a number of Pearson correlations were carried out between all the dependent variables (psychological well-being and MHQoL-7D scoring) in order to evaluate the hypothesis that the dependent variables would be correlated with one another in the moderate range. The findings of the correlation test revealed that there was a significant pattern of relationships among all of the dependent variables (all correlations were positive and in the moderate range; p < 0.01), suggesting MANOVA was appropriate. The Box’s M value of 14.17 was associated with a P value of .028. Based on Huberty and Petoskey’s guidelines, this was considered nonsignificant (i.e., P < 0.005). Therefore, it was presumed that the covariance matrices between the groups were equal to the objectives of the MANOVA.

MANOVA was conducted to test the hypotheses Table 5; for the hypothesis that mean differences would exist between the three levels of independent variables (left, right, and ambidextrous) with regard to the dependent variables (quality-of-life score and psychological well-being). The MANOVA effect does not show a statistically significant association between handedness distribution and either the quality of life (P=0.140) or psychological well-being (P=0.066) scores. Thus, the hypothesis was rejected.

**Table 5:** Summarization of association with handedness distribution regarding quality-of-life score, and psychological well-being, in Saudi Arabia.

P	Std.Deviation	Mean	N=2826	Handedness distribution	Dependent variables
0.140	3.80937	14.9824	908	Left-handed	Quality of life score
	3.65798	15.5698	1727	Right-handed	
	3.62938	15.1630	227	Ambidextrous	
0.066	2.36429	6.6520	908	Left-handed	Psychological well-being
	2.41363	7.0869	1727	Right-handed	
	2.56652	6.8987	227	Ambidextrous	

Then, the data was used to run the MANCOVA (Multivariate Analysis of Covariance) test, which removes the effects of more than one covariate from the model. Covariates such as age, gender, marital status, region, educational level, occupation, and medical history were added to the model. This allows us to see the true effect of the independent variables on dependent variables without unwanted interference. After analysis relationships were also not found between the three levels of independent variables (left, right, and ambidextrous) with regard to the dependent variables (quality-of-life score and psychological well-being). Other covariates that recorded a significant effect on both quality of life and psychological well-being were age, gender, medical status, and medical history of chronic conditions (Table 6). Therefore, it is not necessary to perform an alpha correction, such as a Bonferroni correction, to take into account the running of numerous ANOVAs.

**Table 6:** Summary of the effect of covariates of association between handedness distribution regarding quality-of-life score, and psychological well-being, in Saudi Arabia.

Covariate		P
Age	Quality of life score	0.004
	Psychological well-being	0.001
Gender	Quality of life score	0.004
	Psychological well-being	0.025
status	Quality of life score	0.001
	Psychological well-being	0.001
Education	Quality of life score	0.003
	Psychological well-being	0.142
Occupation	Quality of life score	0.001
	Psychological well-being	0.109
Medical history of chronic conditions	Quality of life score	0.001
	Psychological well-being	0.001
Region	Quality of life score	0.573
	Psychological well-being	0.771
Nationality	Quality of life score	0.096
	Psychological well-being	0.376
Handedness	Quality of life score	0.140
	Psychological well-being	0.066

## Discussion

By assessing a sample of the adult population (N = 2862) in every region of the Kingdom of Saudi Arabia, the current study sought to gauge the prevalence of left-handedness and associated preferences as well as to ascertain the impact of left-handedness on quality of life and wellness. According to the study’s findings, there were 1727 people who were right-handed, or 60.3% of the population, compared to 31.7% who were left-handed. Hand preference is a striking difference in human behavior, with 90% of people globally preferring to use their right hand for numerous tasks and just 10% their left. While the average percentage of left-handers among those born outside the UK was 6.8%, it was 10.1% in England and intermediate in the other UK nations based on a large cohort (about 500,000 members) of the UK biobank [15], which is inconsistent with this finding. These regional variations most likely reflect cultural influences. For instance, it's possible that hand-swapping throughout childhood outside of England was more common or persisted for a longer period of time [15].

About 90% of people prefer to use their right hand for complex manual tasks [16]. Regarding the frequency of preference for using the left hand by left-handed participants in various activities, the survey found left-handed participants preferred to use their left hand for most tasks, including writing, drawing, erasing paper, cleaning teeth, combing hair, catching a ball, and taking cards off the table, consistent with their personal strength. However, just 42.5% of people favored left-handedness when holding a spoon, whereas 57.5% chose right-handedness. Our findings were in line with an earlier study [2]; the study found that most left-handed people preferred to use their left hand for most tasks, such as writing, brushing their teeth, and combing their hair, with 86.24%, 97.28%, and 84.62% correspondingly, more often than their right hand. However, only 35.84% of people preferred using their left hand when holding a spoon, while 64.14 % preferred their right hand [2]. This could be a result of parents encouraging their children to use their right hand for tasks like eating or writing, or it could simply be that religion has a strong influence and discourages using the left hand for tasks like eating, drinking, and conducting religious rites. In China attitudes and behaviors regarding left-handers have been and continue to be influenced by over-determined factors that, while transcending particular cultures, also function as a reaction to historical and cultural influences. Many people in North and East Africa, like the Chinese, try to "cure" left-handedness by imposing severe punishments and restrictions. The actual and reported prevalence of left-handedness appears to have decreased as a result of all of these traditional beliefs and pragmatic factors coming together [17].

Results indicated that there were no significant differences between left-and right-handed people in quality of life or psychological well-being scores. However, right-handed people had a 6.1%, better QOL score than left-handed adults. Therefore, “statistical significance” should not be confused with the importance of an effect [18]. Evidence highlighted the significance of taking left-handed people’s demands into account and utilizing technological solutions to reduce the negative consequences of using equipment that is not fit for them [19].

In another cross-sectional study targeting a population in Croatia (N = 686), the quality of life of left-handed people participating in everyday occupations, using different accessories, tools, and methods of carrying out occupations was examined. The findings described the strong societal pressure that the participants experienced as children (particularly the assumption that they write with their right hand), as well as the difficulties they had in their day-to-day jobs. Most participants rated their quality of life as high despite the experiences mentioned, which they attributed to their personal adaptation to the right-handed world in which they live. However, they also stressed the need for a universal design of everyday objects and the environment to ensure equal access regardless of lateralization [20]. One of the most recent studies also tested a sizable Australian population sample (N = 15,376) to ascertain whether left-handedness is still associated with a higher prevalence of mental illness and addictive behavior. Findings showed that mental illness, incarceration, and problem gambling were not more common among left-handers than right-handers [21]. Another data included 132 participants indicated no statistically significant difference in participants' anxiety and locus of control between the left-and right-handed sample; thus left-handed have a higher self-concept than the right-handed sample. [22]. Future research should be directed toward examining the factors that may affect the quality of life of the left-handed population.

Strengths and limitations

According to authors’ knowledge, this is the first nationwide study in the Kingdom of Saudi Arabia that measures the prevalence of left-handed preference and inquires whether left-handedness can affect the quality of life and wellness. Despite the subsample being randomly chosen from a larger population, the group of people used for research might not have been entirely representative of the general population, which may have diminished the accuracy of study findings and, consequently, the capacity to identify some minor effects, especially when interactions are involved. Additionally, using an online survey could have influenced the results in a biased way.

According to the result, right-handed people have a 6.1% better quality of life than left-handed adults; thus, further research in this area is needed with larger sample sizes to assess the difference adequately. Furthermore, if we combine a qualitative study with quantitative research, the result could be more comprehensive.

Further study is needed because of the minority status and gender distribution of people who use their left hand. According to the present research, women made up the majority of left-handed adults. Could it be that females were more likely to respond to the survey and express their needs? Further research should look for reasons for this phenomenon.

## Conclusions

This study’s findings led to the conclusion that 31.7% of the population was left-handed, according to the distribution of people in this survey showed. Additionally, it was discovered that using two hands had no bearing on one’s quality of life or psychological well-being. To further investigate this discovery, more research with a bigger sample size is required.
